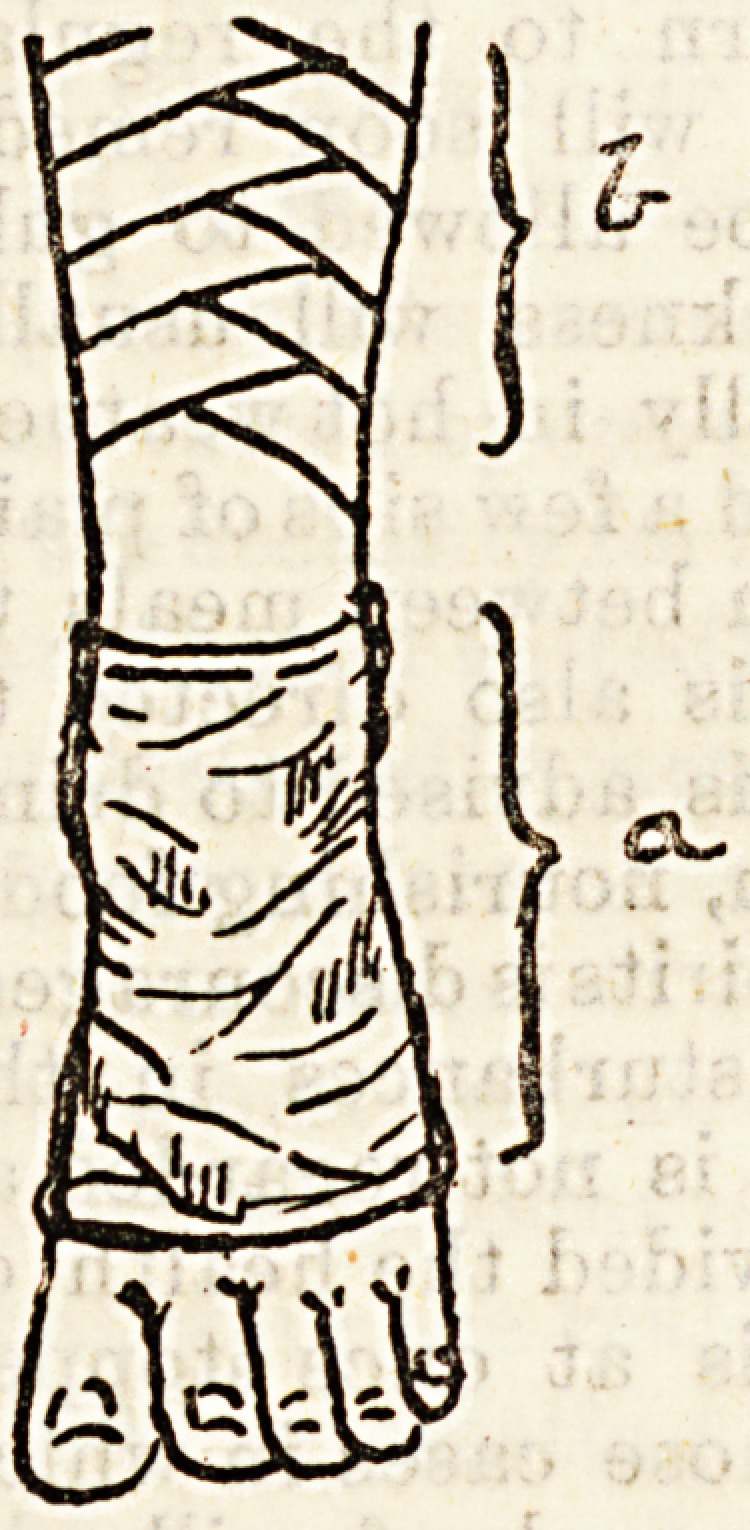# Treatment of Chronic Ulcers of the Leg

**Published:** 1892-11-12

**Authors:** 


### QUEEN'S HOSPITAL, BIRMINGHAM.

### Treatment of Chronic Ulcers of the Leg.

Although presenting many differences as regards their ætiology and pathology, chronic ulcers of the lower limb may fairly be grouped for all practical purposes under one heading, inasmuch as they resemble one another in one most important point—their stubborn resistance to any improvement under the influence of either local or general remedial agents. Occurring for the most part in individuals who are approaching, or who are well past, the middle period of life, and whose vitality has been already lowered through disease, deficient food, and unhealthy surroundings, they form the largest, probably, and, at the same time, the most unsatisfactory class of cases with which we have to deal in the out-patient departments of our hospitals.

Without entering into detailed descriptions of the various forms and conditions met with, it will suffice for the purpose of this article to state that their usual situations are on the outer and front aspects of the leg, between the ankle and the calf, and that they are characterised in the main by abrupt and irregular edges, with the bases dry and flat or covered by feeble granulations, yielding but a scanty serous or purulent discharge, whilst the surrounding skin is congested and indurated, an indication of long-continued passive hyperæmia.

Whether they are the result of traumatism in individuals with feeble circulation and deficient powers of repair or are associated with varicose veins or a specific taint, is of little moment, for their chronicity arises from identical causes, and their successful treatment is dependent on the removal of these factors which delay the process of healing, and which are—

(1) Constant irritation and movement of the parts, whereby the edges of the sore are dragged apart, and the granulations destroyed.

(2) The induration has anchored the surrounding skin to the subjacent fascia and bone, and thus the contraction which is essential to healing is prevented.

(3) Low vitality of the granulating surface.

By far the most potent cause, and, at the same time, the most difficult one to overcome is the movement of the part, and that treatment which holds out the greatest hope of success is absolute rest; for the more complete the rest the more rapid and permanent is the cure.

Unfortunately, however, with the majority of the patients with whom we have to deal such a course of rest would be equivalent to starvation, and therefore we are compelled to devise means whereby the greatest possible amount of support and rest may be obtained whilst enabling the unfortunate sufferers to pursue some form of employment sufficient to provide them with the necessaries of life. For this object many methods have been resorted to, and include many varieties of bandages and supports; but the method which has met with the most satisfactory results, and is almost uniformly adopted at the Queen's Hospital is that which was suggested by Baynton, namely, strapping the affected limb, and the details of which will now be discussed.

The properties of strapping are (1) it acts as a support, (2) exerts pressure, (3) prevents movement.

As a preliminary to strapping, it is advisable to prepare the ulcer so as to clear the surface of any foul discharge, and soften the surrounding tissues if at all hard and dry. The ulcer, therefore, should be dressed for 24 hours, sometimes longer, with some antiseptic wet dressing, and the one usually employed is lint soaked in a saturated solution of boracic acid and sufficiently extensive to cover not only the ulcerated surface but a good extent of the surrounding skin; over this is placed a piece of gutta percha tissue to prevent evaporation, and this is secured by a covering of wool and a bandage. As soon as the ulcer has been sufficiently cleansed and softened by this means, the strapping is to be applied, either directly, or the ulcerated surface may be protected by a piece of lint of corresponding size smeared with ointment, such as unguentum iodoformi or unguentum hydrargyri oxidi rubri diluted with two parts of simple ointment.

The strapping should be cut into strips 1½ inches broad, and equal in length to one and a-third times the circumference of the part to be strapped. Although in the simpler cases it may be sufficient for the strapping to extend from two inches below to two inches above the ulcer, yet it is advisable in most cases to include the ankle, and where the surface is subjected to much movement to fix also the knee.

In strapping the first strip is fixed to the back of the heel, just above the os calcis, and is brought round to the front of the ankle and crossed; the second strip is passed beneath the sole of the foot, at the root of the toes, and brought over the dorsum, so as to cross over the ends of the first piece, and so on. To aid in supporting the limb there may now be applied some cotton wool and bandages. It is advisable to restrap the limb every third day, if possible, at the commencement of treatment, and then once a week when the healing process has been well started. The strapping must be continued until the whole surface has healed.

Constitutional remedies may be used when indicated as subsidiary agents, and, as far as possible, the limb should be elevated and rested.

Another method which is sometimes adopted, and with marked success, is the fixing of the ankle by a plaster of paris case (Fig. *a*), which can remain undisturbed during the whole period of treatment, whilst the ulcerated surface and its more immediate neighbourhood is strapped (Fig. *b*).

This method has two advantages over the strapping alone, as it economises the time and material which the continual restrapping of the ankle would entail, and it, moreover, acts as a far stronger support to the ankle joint in those cases where the patient is compelled to stand. In some cases it is an advantage to fix the knee joint by a similar plaster collar.

Either method, if carefully carried out, will succeed when all other means have failed, and should the patient remain unbenefited there is nothing left but complete rest in bed.

## Figures and Tables

**Figure f1:**